# A meta-analysis of deltoid ligament on ankle joint fracture combining deltoid ligament injury

**DOI:** 10.3389/fsurg.2023.976181

**Published:** 2023-03-27

**Authors:** XiaoLing Yang, Jianshuang Zeng, Wei Yang, Ronnell D Dela Rosa, Zhixia Jiang

**Affiliations:** ^1^ Department of Nursing, Guizhou Nursing Vocational College, Guiyang, People's republic of China; ^2^ School of Nursing, Philippine Women's University, Manila, Philippines; ^3^ Department of Dermatology, Guizhou Provincal People's Hospital, Guiyang, People's republic of China; ^4^ Department of Neurosurgery, People's Hospital of Dechang County, Dechang, People's republic of China; ^5^ College of Nursing and Midwifery, Bataan Peninsula State University, Balanga, Philippines

**Keywords:** meta-analysis, ankle fracture, deltoid ligament injury, ligament repair, ligament reconstruction

## Abstract

**Purpose:**

Ankle fracture combined with deltoid ligament (DL) injury results in decreased stability of ankle mortise, reduced contact surface between tibial and talus, increased local stress, and increased postoperative complications. The purpose of this meta-analysis was to evaluate the postoperative effects of repairing ligaments in ankle fractures with DL rupture.

**Methods:**

According to the steps of the Cochrane systematic review, the related literatures from PubMed, Embase and the Cochrane Library Databases were retrieved as of September 1, 2021, and all relevant randomized controlled trials and retrospective studies were collected. The evaluation indicators include medial clear space (MCS), visual analogue scale (VAS), American Orthopedic Foot and Ankle Society (AOFAS), complications rate. Meta-analysis was conducted by RevMan® 5.3 provided by the Cochrane collaboration.

**Results:**

A total of 388 patients (195 patients in the ligament repair group and 193 patients in the non-repair group) were included in 7 clinical trials. Meta-analysis data showed there were no statistically significant differences between the ligament repair group and non-repair group in final follow-up VAS, final AOFAS and postoperative MCS (*P* = 0.50, *P* = 0.04, *P* = 0.14, *P* = 0.14, respectively). Final follow-up MCS and complications rate in ligament repair group were smaller than those in the non-repair group and were statistically significant (*P* < 0.00001, *P* = 0.006, respectively).

**Conclusion:**

Although there was no difference in in final follow-up VAS, final follow-up AOFAS and postoperative MCS between experimental group and control group, It's statistically significant in final follow-up MCS and complications rate. Ligament repair could reduce the width of MCS, restore ankle stability, reduce the incidence of complications and lead to a better prognosis.

## Introduction

Ankle fracture is one of the most common lower limb fractures, accounting for about 3.92% of total body fractures. According to Hintermann et al., about 40% of ankle fracture patients were accompanied by partial or complete deltoid ligament (DL) repture (1). In an MRI study conducted by Jeong MS et al. (2), the proportion of DL partial or complete Deltoid ligament (DL) fracture was up to 58.3%. The DL complex is anatomically a series of ligaments on the medial side of the ankle. Since Milner and Soames first proposed that the DL was divided into superficial and deep layers (3), the concept of shallow and deep layers has been widely accepted. The accompanying biomechanical studies have also gradually proved that the deltoid ligament is the main structure of maintaining the stability of the talus. The DL is more important than the lateral malleolar ligament in maintaining ankle joint stability (4, 5). The main function of the deltoid ligament is to keep the ankle joint stable (6, 7). Therefore, clinically, many people believe that failure in timely treatment of DL injury will lead to atrophy of the broken ligament end, and then result in decreased tension of the DL, unstable ankle, swelling and pain of the medial malleolus, and increasing the risk of traumatic arthritis (8, 9). However, Some studies (10, 11) suggest that fractured DL can heal spontaneously without surgical intervention. Surgical exploration and repair of ruptured ligament would not only increase the operation time, causing soft tissue injury, prolonging the time of operation and Postoperative recovery, but also have no significant difference in the incidence of postoperative complications compared with conservative treatment.

**Table 1:** Summary of included studies.

Source	Country	Design	Total No. of patients included (Repaired/unrepaired)	Mean Age, y	Outcome measures analyzed	Mean follow-up period
Plazas et al, 2011 (14)	Brazil	Retrospective Level III	44 (33/11)	Treatment: 42 Control: 41	AOFAS, complications	12
Wu et al, 2017 (15)	China	Prospective Level II	48 (22/26)	Tota:39.6	AOFAS, VAS	22
Gu et al, 2017 (16)	China	Prospective Level II	40 (20/20)	Treatment: 40.6 Control: 37.5	AOFAS, VAS, complications	12
Zhao et al, 2017 (17)	China	Retrospective Level III	74 (20/54)	Total: 39.5	AOFAS, MCS, VAS, complications	14
Woo et al, 2017 (18)	Korea	Retrospective Level III	78 (41/37)	Treatment: 41.6 Control: 39.4	AOFAS, MCS, VAS, complications	12
sun et al, 2018 (19)	China	Retrospective Level III	41 (28/13)	Treatment: 35.5 Control: 30.5	MCS, AOFAS	40
Chen et al, 2020 (20)	China	Retrospective Level III	63 (31/32)	Treatment: 53.7 Control: 52.9	AOFAS, MCS, VAS, complications	12

So far, there are not enough clinical studies with large samples and long-term follow-up to reach a consensus on this issue. Therefore, this Meta-analysis aims to conduct a meta-analysis based on the results of relevant published clinical trials and follow-up data to preliminarily evaluate whether surgical treatment or conservative treatment is more beneficial to patients in terms of long-term prognosis, so as to provide reference for clinicians in treatment planning.

## Materials and methods

This study was conducted according to the Systematic Reviews and Meta-Analyses (PRISMA) guidelines (12). The two investigator independently searched pubMed, Embase, and the Cochrane Library databases for relevant studies using the following keywords and their synonyms:(“ankle” AND “fracture” AND “deltoid”). The deadline is 2021–09–01. References cited in previous published meta-analyses and review articles were added manually. They independently reviewed all the abstracts and contents of the article abtained in the previous steps.

### Study eligibility criteria

Inclusion criteria:1. Comparative studies on the outcome of DL repair in acute ankle fracture; 2. One of the following outcome is reported: postoperative MCS, final follow-up MCS, final follow-up VAS, 3. Final follow-up AOFAS, final follow-up complication rate; 3. Mean follow-up time ≥12 months.

Exclusion criteria: 1. Duplicate literature; 2. Letters, conference reports, reviews, reviews and unproofread papers; 3. The AOFAS score is not presented as continuous numerical form.

### Risk of bias assessment

Literature quality evaluation was independently completed by the two investigators according to The Newcastle-Ottawa Quality Assessment scale (13).

### Data extraction

The first author's name, publication time, countries, the number of cases, control group, follow-up period, postoperative MCS, final follow-up MCS, final follow-up VAS, final follow-up AOFAS and complication rates were extracted from the literature by the two investigators independently. If the results are inconsistent, a consensus shall be reached after joint research.

### Data analysis

Data were analyzed by Revman 5.3. For continuous variables, standardized mean difference (SDM) and 95% confidence interval (CI) were calculated. For dichotomous variables, the relative risk (RR) and 95% confidence interval (CI) were calculated. Heterogeneity was tested using Cochran's *Q* test and I² test provided by Revman 5.3. If *P* (Cochran's *Q* test) > 0.1 and *I*² < 50%, the fixed-effect model is selected. When *P* (Cochran's *Q* test) < 0.1, *I*² > 50%, the random effect model is selected.

## Results

**Figure 1:**
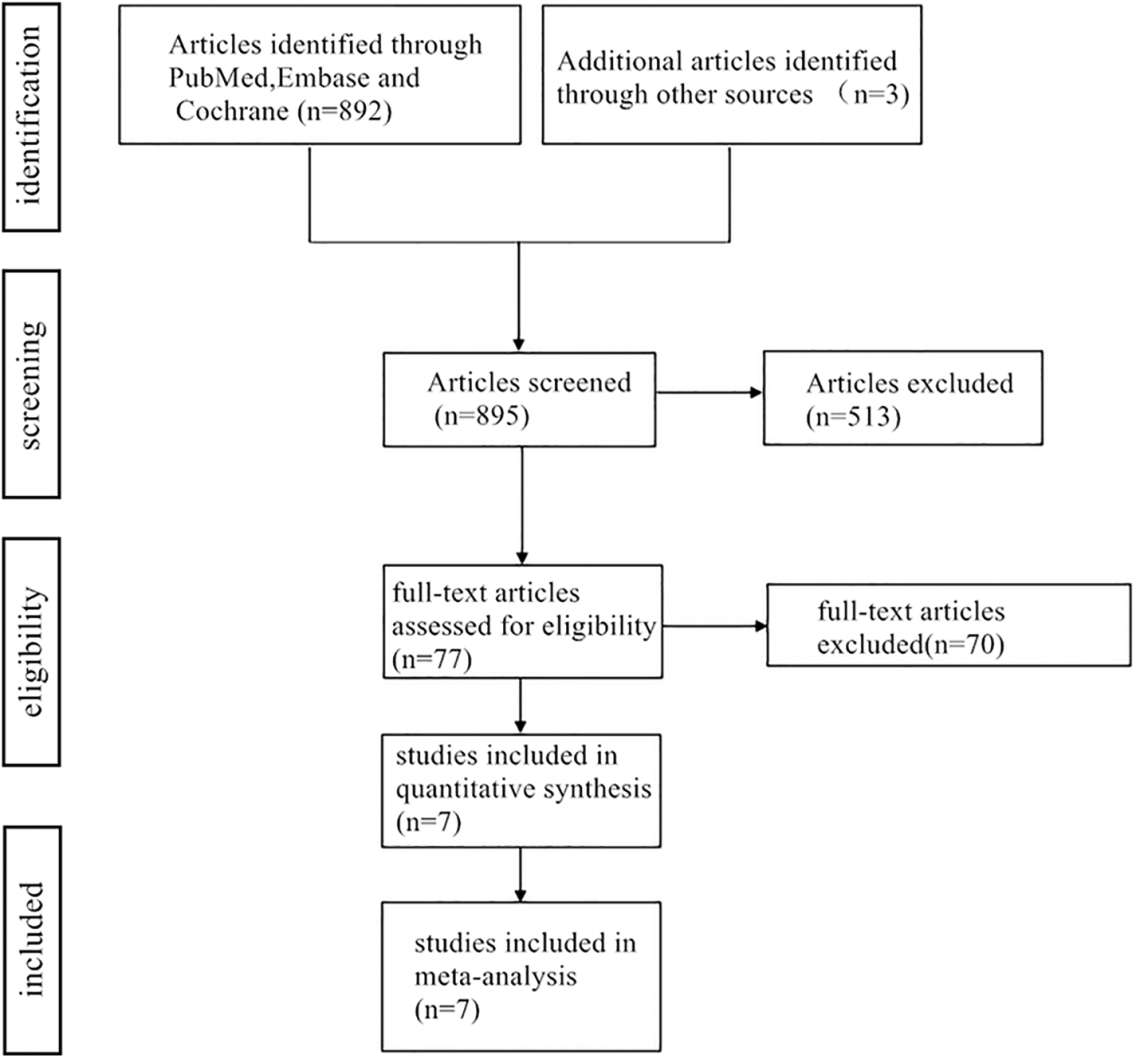
PRISMA chart.

A total of 895 articles were retrieved from Pubmed, Embase, the Cochrane Library databases. 305 duplicate articles were excluded by Endnote software and 513 irrelevant articles were excluded by reading the title and literature abstract, the final number was 77. A total of 388 patients (195 patients in the ligament repair group and 193 patients in the non-repair group) were included in 7 clinical trials (Figure 1).

### Demographics

A total of 388 patients (195 patients in the ligament repair group and 193 patients in the non-repair group) were included in 7 clinical trials. The mean age was 42.2 years in the repair group and 41 years in the non-repair group. Mean follow-up of all subjects was 16.6 months.

### Quality assessment

Table 2 summarizes the results of the different domains of study quality, as adapted from the Newcastle–Ottawa Scale (13). All studies were judged on eight items and categorized into three groups: selection of study groups, comparability of groups, and assessment of the outcome of interest. A total of nine stars deemed the study to be of the highest quality.

**Table 2:** Newcastle-Ottawa quality assessment of the included studies in the meta-analysis.

Domain	Item	Plazas et al	Wu et al	Gu et al	Zhao et al	Woo et al	Sun et al	Chen et al
Selection	Representativeness of the exposed cohort	*	*	*	*	*	*	*
	Selection of the non-exposed cohort	*	*	*	*	*	*	*
	Ascertainment of exposure	*	*	*	*	*	*	*
	Demonstration that outcome of interest was not present at start of study	*	*	*	*	*	*	*
Comparability	Comparability of cohorts on the basis of the design or analysis	*	*	*	*	*	*	*
Outcomes	Assessment of outcome	*	*	*	**	**	**	**
	Was follow-up long enough for outcomes to occur	*	*	*	*	*	*	*
	Adequacy of follow-up of cohorts	*	*	*	*	*	*	*
Total score		8	8	8	9	9	9	9
Result		Good	Good	Good	Good	Good	Good	Good

### Results of individual studies

#### Postoperative MCS

**Figure 2:**
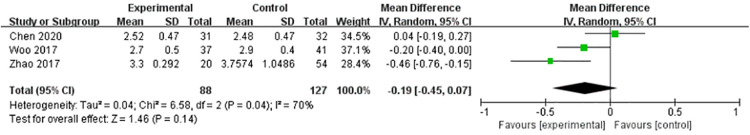
No statistical difference between experimental and control group (WMD = 0.19, 95% CI = [0.45, 0.07], *P* = 0.14).

Three articles provided comparative data on postoperative MCS. Heterogeneity test showed moderate heterogeneity in this analysis (*P* < 0.1, *I*^2 ^= 70%). Fixed effect model (FE) was adopted. There was no statistical difference between experimental group and control group (WMD = 0.19, 95% CI = [0.45, 0.07], *P* = 0.14). It showed that there was no significant difference in Postoperative MCS between the two groups (Figure 2).

#### Final follow-up MCS

**Figure 3:**
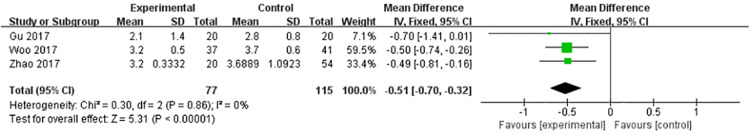
Final follow-up MCS could be significantly reduced in the experimental group (WMD = 0.51, 95% CI = [0.70, 0.32], *P* < 0.00001).

Three articles provided comparative data on final follow-up MCS. Heterogeneity test showed that there was no heterogeneity in this analysis (*P* > 0.1, *I*^2 ^= 0%). Fixed effect model (FE) was adopted. There was statistical difference between the two groups in final follow-up MCS (WMD = 0.51, 95% CI = [0.70, 0.32], *P* < 0.00001). Final follow-up MCS could be significantly reduced in the experimental group (Figure 3).

#### Final follow-up VAS

**Figure 4:**
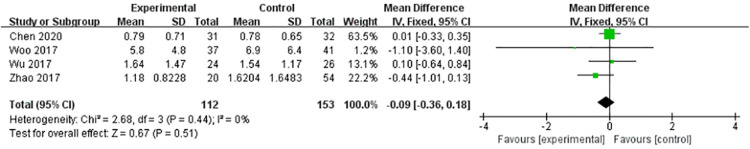
No statistical difference between experimental group and control group (WMD = −0.09, 95% CI = [-0.36, 0.18], *P* = 0.51).

Four articles provided comparative data on Final follow-up VAS. Heterogeneity test showed that there was no heterogeneity in this analysis (*P* > 0.1, *I*^2 ^= 0%). Fixed effect model (FE) was adopted. There was no statistical difference between experimental group and control group (WMD = −0.09, 95% CI = [-0.36, 0.18], *P* = 0.51). Final follow-up pain scores did not differ between the two groups (Figure 4).

#### Final follow-up AOFAS

**Figure 5:**
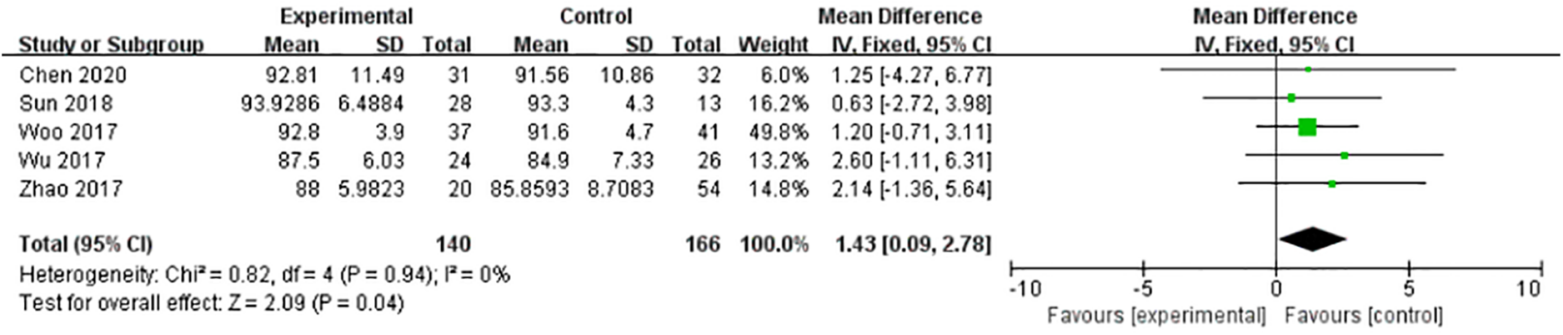
No statistical difference between experimental group and control group (WMD = 1.43, 95%, CI = [0.09, 2.78], *P* = 0.04).

Five articles provided comparative data on Final follow-up AOFAS. Heterogeneity test showed that there was no heterogeneity in this analysis (*P* = 0.94 > 0.1, *I*^2 ^= 0%). Fixed effect model (FE) was adopted. There was no statistical difference between experimental group and control group (WMD = 1.43, 95%, CI = [0.09, 2.78], *P* = 0.04). Final follow-up functional outcomes did not differ between the two groups (Figure 5).

#### Complications rate

**Figure 6:**
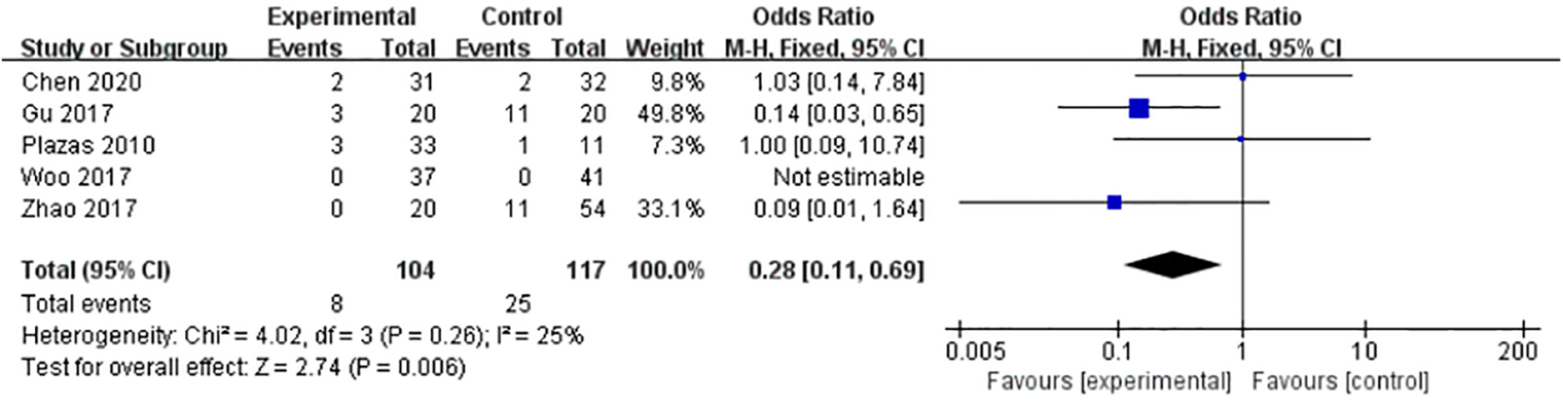
Combined deltoid ligament repair could reduce the incidence of postoperative complications (OR=0.28, 95% CI = [0.11, 0.69], *Z* = 2.74, *P* < 0.01).

Five articles provided comparative data on complications rate. Heterogeneity test results (*P* > 0.1, *I*^2 ^= 25%) suggested mild heterogeneity. Fixed effects model (FE) was adopted. Meta-analysis suggested statistical significance between the two groups (OR=0.28, 95% CI = [0.11, 0.69], *Z* = 2.74, *P* < 0.01). The results showed that combined deltoid ligament repair could reduce the incidence of postoperative complications (Figure 6).

## Discussion

In this meta-analysis, there was no statistical difference in postoperative MCS, final follow-up VAS and final follow-up and AOFAS. But it was statistically significant on final follow-up MCS and complications rate. The results showed that adding ligament repair could reduce final follow-up MCS and complications rate. Anatomical and biomechanical mechanisms of this result was that deltoid ligament repair could reduce MCS, ankle joint contact area, weight-bearing stress and complication rate.

Theoretically, the theoretical basis of repair and non-repair comes from the circle theory and the repair theory. In 1953, Neer proposed the ankle ring theory for the first time (14) believing that the bone structure and soft tissue structure of the ankle joint together constitute a closed ring. When there is only one gap in the ankle ring, other structures will compensate to keep the ring stable. And only when two or more structures are injured, it would result in unstability of ankle ring. Based on this theory, for ankle fracture with deltoid ligament injury and not with medial malleolus injury, the ankle joint stability could be obtained without repairing the deltoid ligament after combined reduction and fixation of lateral malleolus and inferior tibiofibular joint. But the repair theory holds that the ring theory is limited to the stability in coronal plane, the ankle joint is still unstable in the sagittal plane, so the overall stability cannot be abtained. Therefore,ligament repair should be combined with anatomical reduction and fixation of the bony structure to achieve ankle joint stability.

Although there are still controversies about the anatomical composition and variation of the deltoid ligament, it is generally believed that the deltoid ligament can be divided into two layers: superficial and deep components. With the in-depth research of biomechanics in mortise-joint ligament, the previous belief that the lateral collateral ligament is more important than the deltoid ligament in maintaining joint stability has been changed (15, 16). By applying axial stress in biomechanical study to simulate the stress of normal ligament under loading condition, it is considered that the trigonal ligament is more important for maintaining ankle joint stability (4, 5, 17). Currently, the deltoid ligament is believed to serve two major functions, stabilizing the ankle joint and conducting tibial motion. The most important of which is maintaining stability of the medial malleolus (6, 7, 18, 19). Most experts believe that the deep deltoid ligament is more important than the shallow ligament in maintaining ankle stability. Most experts agree that the deeper layer is more important than the superficial layer in maintaining ankle stability. Klein et al. (20) found that when the superficial layer was ruptured or torn, the displacement of the talus was not obvious, and the stability of the ankle joint did not change significantly. When the superficial and deep components were ruptured or torn together, the inclination Angle of the talus could reach up to 14°, and the ankle joint was seriously unstable. Rasmussen et al. (21) found that the role of the posterior tibial deep ligament is mainly to limit dorsiflexion of the foot and the function of the anterior deep tibial pitch ligament is to limit toe flexion. Other scholars also conducted some biomechanical studies on the functions of various parts of the deltoid ligament. For example, Earll et al. (22) found that after the cut off of tibial ligament, the contact area of tibial talus joint was reduced by 26%–43%, the joint surface pressure of the distal tibia was increased by 20%–30%, the center of gravity was deviated outward, and the medial malleolus space was widened. No significant changes were observed when other ligaments were cut off. Milner (23) found that the middle calcanoscaphoid ligament, the tibioscaphoid ligament and the deep posterior tibiotalus ligament are the most important ligaments for ankle joint stability.

The earliest support for deltoid ligament repair came from Johnson and Hill in 1988 (24), who reported that over 60% of patients had residual tenderness of the medial ankle after surgery, and 38% had persistent medial instability one year later. Subsequently, Hintermann B et al. (25) reported that repair of the deltoid complex with suture anchors showed good to excellent scores in AOFAS in over 90% of the patients. Deltoid ligament repair can reduce the incidence of MCS widening. In 2010, Plazas et al. (26) retrospectively analysed the AOFAS scores of patients with deltoid ligament repair and those without repair one year after surgery, and the results showed that the operative group was superior to the non-operative group. Horisberger (27) and colleagues noted that traumatic arthritis reached 20.4% among hospitalized patients without deltoid ligament repair. In 2015, Yu et al. (28) published a multicentre study. All AOFAS, VAS and Short Form-36 scores improved significantly after repair and it showed deltoid ligament repair in patients with an unstable medial ankle after fracture fixation could prevent ankle stabilization-related complications. Zhao et al. (17) retrospectively analyzed 74 cases, 20 of which were repaired surgically and 54 of which were not repaired, measured AOFAS score, VAS score,preoperative MCS, postoperative MCS and final follow-up MCS. They concluded that deltoid ligament repairing could help reducing the incidence of postoperative poor reduction, especially in type C ankle fracture of AO classification. Chen et al. (29) retrospectively analyzed the outcome of 63 patients with supination-external rotation stage IV ankle fracture combining with deltoid ligament rupture. The deep deltoid ligament repair group (31 patients) showed no significant difference from the nondeep deltoid ligament repair (32 patients) in radiographic performances,such as talus Angle (TA), fibula length (FL), medial tibial malleolus Angle (TMMA), medial transparent space (MCS) and tibial fibula transparent space (TFCS). But in the comparison of postsurgery functional outcomes, The deep deltoid ligament repair group had significantly reduced VAS score (*P* < 0.05), with markedly increased RMBA (*P* < 0.05) compared to thenondeep deltoid ligament repair. Chen et al. believed that ligament repair was more advantageous in SER stage IV fracture with deltoid ligament rupture.

Although there is considerable anatomical, biomechanical and clinical evidence to support deltoid ligament repair in ankle fracture. There is still a contrary view in clinical practice. In 1997, C Maynou et al. (30) compared the follow-up results of 18 patients in the surgical repair group with 17 patients in the non-surgical repair group. Roentgenograms including A.*P*, lateral, mortise view and a external rotation stress view described by Kleiger showed no significant difference between the two groups, likewise no differences were observed for postoperative complications rate. Subsequently, Stromsoe et al. (10) conducted a randomized double-blind clinical trial in 1998, in which 50 patients with Weber type B and C ankle fractures were randomly divided into two groups: repair group and non-repair group. At the end of follow-up, working ability, Sports activities, postoperative complications and other related indicators showed no significance between the two groups. Wu et al. (31) compared the outcomes of transsyndesmotic fixation to DL repair with suture anchor. No statistically significant differences were found in the AOFAS score, SF-36 score, or VAS score between the 2 groups. In 2017 Woo et al. (32) retrospectively evaluated 78 consecutive cases of a ruptured DL with an associated ankle fracture. Patients in the conservative treatment for ruptured DL underwent management from 2001 to 2008 (37 fractures, group 1), while the operative treatment for ruptured DL was included from 2009 to 2016 (41 fractures, group 2). The clinical outcomes were not significantly different between the 2 groups. In 2018 Sun et al. (33) conducted a prospective comparative cohort study to determine whether it is necessary to routinely repair the injured DL. They compared Philips and Schwartz Score, MCS and AOFAS among different groups. The average follow-up time was over 40 months. In conclusion, the results of this study do not support routine repair the injured DL.

After the triangular ligament is damaged, the coronal closed loop of the ankle joint is destroyed, the stability of the medial side of the ankle joint is reduced, and the force line of the rear foot gradually changes, followed by related deformities such as flat feet, which will lead to the occurrence of cartilage degeneration in the ankle joint in the long run. The medial malleolus injury can be confirmed by comprehensively combining the patient's symptoms, imaging data, and ankle arthroscopic exploration. Whether to perform surgical intervention on acute deltoid ligament injury is still controversial. Diagnosis and treatment options are different for different types of ankle joint injuries. Due to the possible occurrence of inferior tibiofibular screw fixation, the academic community is exploring the use of triangular ligament repair to replace lower ankle joint injuries. Tibiofibular screw fixation can avoid problems such as secondary surgery and poor tibiofibular reduction. Several studies have reported outcomes after repair of the deltoid ligament in patients 270 with ankle fractures. YU et al. (34) conducted a multicenter retrospective study of 131,271 patients after deltoid ligament repair, and found no widening of the medial malleolus 272 space or increased valgus tilt angle at the final follow-up. ZHAO et al. (35) 2 compared 273 patients who underwent deltoid ligament repair with those who did not. After a mean 274 follow-up of 53.7 months, they found that the non-repair group had a poor reduction 275 rate of 20.4%, while the repair group had no malreduction. SHEN et al. (36) reported 276 the imaging and clinical results after the repair of the deltoid ligament with suture anchors. They found that the medial malleolus space was well maintained after the 278 operation, and the clinical effect was satisfactory. Sherif Dabash's study (37) points out 279 that Current literature does not provide clear indication for repair of the deltoid ligament 280 at the time of ankle fracture repair. There may be some advantages of adding deltoid 281 ligament repair for patients with high fibular fractures or in patients with concomitant 282 syndesmotic fixation. Indications for operative repair of deltoid ligament (DL) injuries in ankle fracture patients are debated. The current management strategy of most scholars is to explore 285 and repair the deltoid ligament in the following multiple situations: that is, when the patient has ankle dislocation or the medial malleolus space is significantly widened on the stress x-ray film, tissue embedded in the medial malleolus space prevents the reduction of the talus, or External rotation or valgus instability of the ankle still exists 2 after anatomical reduction and fixation of the lateral malleolus; and complete rupture of the deltoid ligament confirmed by arthroscopy.

Limitations of this paper are as follows:

1. Small sample size, including only 7 article with a total of 388 patients resulting in reduced statistical performance; 2. Lack of randomized controlled trials; 3. Heterogeneity of included studies exists, such as differences in ligament repairing methods and proficiency in different institutions.

## Conclusion

Although this meta-analysis showed statistical differences in long-term MCS and complication rates between the surgery group and the control group, it suggested that Ligament repair could reduce the width of MCS, restore ankle stability, reduce the incidence of complications and lead to a better prognosis. But to actually establish a broad consensus on 3 this issue, multicenters RCTs are needed to confirm it.

## Data Availability

The original contributions presented in the study are included in the article/Supplementary Material, further inquiries can be directed to the corresponding author/s.
